# Resignation of a Dispensing Staff

**Published:** 1911-07-01

**Authors:** Robt. H. Mushens

**Affiliations:** Secretary. Sunderland Provident Dispensary


### "RESIGNATION OF A DISPENSING STAFF."

Sir,—In your issue of June 24 there appears a paragraph under the above heading. I suppose "Dispensary Staff," and not "Dispensing Staff," was intended—that, however, is a detail. The paragraph in question states that "in spite of the protests of the medical staff, the management of the Sunderland Dispensary has continued the distribution of a leaflet setting forth the advantages of membership of the Dispensary, and last week things were brought to a head by the resignation in a body of the twelve members of the medical staff."

Would you allow me to say most emphatically that such a statement, which is absolutely untrue and most damaging to the interests of the Sunderland Provident Dispensary as a public institution, cannot be allowed to pass unnoticed. As a matter of fact, there has never been one word of protest made by any member of the medical staff to the issue of the leaflet in question, and had it been objected to, the Committee, which, by the way, includes every member of the medical staff, would have instantly stopped any further issue.

Further, that though the whole of the medical staff did resign, the majority did so under protest.

I shall esteem it a favour if you will give this correction as prominent a position as you gave to the misleading paragraph.—I am, yours truly.

Robt. H. Mushens, Secretary.

Sunderland Provident Dispensary, June 24.

[Mr. Mushens' letter is not clear. He admits that the dispensary staff did resign, as we said, though apparently the decision to do so was not unanimous. The point appears to be that the management of the Sunderland Dispensary has withdrawn the leaflet complained of, and that the action of the medical staff in resigning was not due, as we were informed, to its issue after remonstrance, but to the staff's sudden fear that the British Medical Association would construe the leaflet as an "unprofessional" form of advertisement. This fear was caused by what may be called a hostile resolution on the part of the local branch of the Association on the subject of the management of the Dispensary. The staff's resignation, then, was merely a formal method of defence. The Dispensary Committee, we understand, has pointed out that the notice construed as canvassing "has been in circulation for twenty years," and as regards the circular some sympathy will be felt with Mr. Mushen's apt reminder to the Committee that "advertising in some shape or form is necessary, and I venture to say that there is no hospital or medical institution in the country but does it. . . . Why, the doctor himself advertises. Why does he seek a position as a member of the honorary staff of a hospital? It is pretty largely a matter of self-advertisement." In short, it is the local branch of the British Medical Association which appears to have caused all the trouble.—Ed. The Hospital.]

